# A pilot study of dimeticone oils versus sodium carbonate treatment for tungiasis: A randomized cohort trial in Homa Bay County, Kenya

**DOI:** 10.1371/journal.pntd.0012341

**Published:** 2024-07-23

**Authors:** Kana Suzuki, Yasuhiko Kamiya, Chris Smith, Satoshi Kaneko, Gordon Okomo, Asiko Ongaya, Evans Amukoye

**Affiliations:** 1 School ofTropical Medicine and Global Health, Nagasaki University, Nagasaki, Japan; 2 Department of Clinical Research, Faculty of Infectious and Tropical Diseases, London School of Hygiene and Tropical Medicine, London, United Kingdom; 3 Department of Ecoepidemiology, Institute of Tropical Medicine, Nagasaki University, Nagasaki, Japan; 4 Ministry of Health Homa Bay County, Homa Bay, Kenya; 5 Kenya Medical Research Institute, Nairobi, Kenya; Uniformed Services University: Uniformed Services University of the Health Sciences, UNITED STATES OF AMERICA

## Abstract

**Background:**

Tungiasis is a cutaneous parasitosis caused by the female flea *Tunga penetrans*. Two-component dimeticone (NYDA) is the only treatment for tungiasis recommended by the World Health Organization; however, this topical drug is not available in Kenya. In Western Kenya, sodium carbonate is commonly used in the treatment of tungiasis. This study evaluated the 7-day cure rates for tungiasis by comparing sodium carbonate and NYDA treatments in Homa Bay County, Kenya.

**Methodology/Principal findings:**

This was a randomized, observer-blinded, parallel-treatment cohort trial. Twenty-three eligible children with 126 flea infections were matched and randomized. All participants received both treatments, with one treatment on each foot. We recorded all health conditions/information, including inflammation scores and adverse events. Observations were performed on days 3, 5, and 7 using a digital microscope to confirm dead or live fleas based on the viability signs. Twenty-three children aged 3–13 years were analyzed. The proportion of dead fleas on day 7 was higher after NYDA treatment than after 5% sodium carbonate treatment (87% versus 64%, respectively, *P* = 0.01) NYDA. Median survival was 5 days for both treatments; NYDA had significantly higher trend of flea non-viability rate than 5% sodium carbonate (*P*<0.01). There were no significant differences in the inflammation score or pain/itchiness between the two treatments. On the last day, 14 children indicated their preference for NYDA in future treatment of tungiasis, whereas nine children preferred the 5% sodium carbonate solution.

**Conclusions/Significance:**

NYDA was significantly more effective than 5% sodium carbonate for tungiasis treatment. Both treatments were safe but the children preferred NYDA more. Future studies with more participants and an extended observation period are warranted to confirm our findings. The findings suggest that NYDA should be made more available in tungiasis endemic area.

**Trial registration:**

UMIN-CTR; UMIN 000044320

## Author summary

Jigger infestation is caused by fleas. Most of the recommended treatments for jiggers are unaffordable; thus, local people experience difficulty with the usage. This study contribute to the understanding of the efficacy of two treatments against jigger infestation in the community: a local practice (soda ash solution) and a standard treatment (silicone oil). The study participants were 23 children aged 3–13 years with 126 fleas in Homa Bay County, Kenya. We administered the treatment only on day 1 and followed up on days 3, 5, and 7. At 7 days, the soda ash solution killed 64% of the fleas whereas the silicone oil killed 87% of the fleas. Thus, the safety of both treatments was confirmed. As this is the first step in investigating the efficacy and safety of the soda ash solution, we need to conduct further studies with more participants to compare both treatments.

## Introduction

Tungiasis is a cutaneous parasitosis caused by the female flea *Tunga penetrans* [1], which is distributed in tropical and subtropical regions worldwide. Tungiasis is recognized as a Neglected Tropical Disease skin condition by the World Health Organization (WHO), with the goal of being controlled by 2030 [2].

*Tunga penetrans* are unique within the realm of fleas; non-fertilized females penetrate the skin and remain there until they die in situ after 4 to 6 weeks [3,4]. An estimated 1.4 million (4%) Kenyans suffer from tungiasis [5]. Age-specific prevalence shows a pattern with maximum occurrence in children between the ages of 5 and 14 years and in older adults [6]. Children with tungiasis are reported to have disproportionately high absenteeism from school, and they perform worse in class than unaffected pupils because constant itching and pain make it difficult for them to concentrate [7].

If not managed properly, infestation by *Tunga penetrans* can result in negative health and socioeconomic effects due to the development of wounds/ulcers [2]. Tungiasis is diagnosed by visual inspection, and the live fleas appear as a whitish disc of varying sizes and a dark point in the middle, which darkens over time until becoming entirely black when dead [2]. Surgical extraction of embedded fleas is common but has several shortcomings, such as pain and secondary infection. According to Kenyan guidelines [5], the following are recommended as treatments for tungiasis: antiseptic solutions including Savlon, potassium permanganate, and hydrogen peroxide, followed by petroleum jelly. Topical flea repellants include Deet, coconut oil, neem seed and coconut oil preparations, jojoba oil, and aloe vera extract. Silicone oil (dimethicone) is also strongly recommended as an effective and nontoxic remedy. The trend in the use of therapeutic agents in research has changed significantly in the last 15 years, from oral to topical treatments. Recent research on therapeutic agent types, including naturally derived treatments, compounding methods, and application methods, has focused on increasing the availability of inexpensive treatments without side effects, such as pain and itching, and increasing the quality of life of patients with tungiasis. Several intervention studies have been conducted in Brazil, Kenya, Uganda, and Nigeria [8–11].

Several clinical trials were conducted. However, those are difficult to use sustainably in many regions; ivermectin and metrifonate were found to be ineffective, while dimethicone is unavailable in endemic communities. In Kenya, communities have treated tungiasis with an herbal formula lotion composed of neem seeds and coconut oil [9]. A recent study in Kilifi County by Elson et al. [9] showed that although 20% neem plus coconut oils did not kill more fleas than potassium permanganate within 7 days, the secondary outcomes were better (i.e., reduced acute pathology and higher odds of children being pain-free). Another intervention study [12] was conducted in Muranga County using 100% coconut oil. However, owing to the short observation period of the study, it remained unclear whether the fleas died according to their natural life cycle or because of therapeutic effects. The aforementioned are common local treatments in East Kenya but are not used in Western Kenya because it is far from the ocean. In Western Kenya, the local practice is the use of sodium carbonate, locally referred to as Soda ash, Magadi or Magadi soda. This naturally available chemical has been used in various industries, including glass manufacturing, glazing of cakes, anti-caking agents for pastries, and chemical detergents in homes. Kenyan communities use Magadi to soften green vegetables and meats. It sells 50 g for approximately 10 ksh (0.1 USD) in local markets. One study in 2016 showed its effectiveness in the treatment of tungiasis in Siaya County, Kenya [13]. The study on sodium carbonate did not use practical comparisons but concluded that it has a certain degree of effectiveness as a treatment.

The two-component dimeticone (NYDA) drug is the only treatment for tungiasis recommended by the WHO and is listed in the national guidelines of Kenya [2,5]. A study conducted in Kenya showed that NYDA applied to the feet and up to the ankles killed 78% of the embedded fleas within 7 days [14]. The expense and unavailability of NYDA largely preclude its use in Kenya. Thus, we compared the local Western Kenyan practice of sodium carbonate application with NYDA treatment, by assessing the cure rates of tungiasis in children, who are the key target population of this disease.

The study protocol has been published previously [15]. This pilot study was performed to investigate the percentage of the dead fleas (the cure rates) following tungiasis treatment with either 5% sodium carbonate orNYDA cohorts by using similar methods in Kenyan children prior to the main non-inferior trial. Other objectives are to assess the safety, acute pathology scores, and acceptability in the community.

## Methods

### Ethics statement

This study was approved by the Medical Research Ethics Review Board of Nagasaki University, Japan (reference number: NU_TMGH_2022_177_1). This study was approved by the Scientific and Ethics Review Committee of Kenya Medical Research Institute (reference number: KEMRI/SERU/CRDR/070/4420). Approval was also obtained from The National Commission for Science, Technology and Innovation (reference number: NACOSTI/P/22/19550) of Kenya. This study was registered with UMIN-CTR (UMIN trial ID: UMIN 000044320; reception desk number: R000050621), on May 28, 2021, which is recognised internationally as follows: https://center9.umin.ac.jp/FAQ/en/UMIN-CTR_e/#faq-5_1.

Data were not collected on the names and affiliations of the participants. Participation of school children in this study was totally voluntary. When the participants felt adverse effects/events, we immediately contacted the medical doctor in charge of this study and got his discretion, ex) referred free of charge to the nearest health facility.

Another possible risk was that the child may be teased for having jiggers since this will be revealed to other children in the school by participating in the study. Before selecting participants, we provided information on tungiasis through health promotion so that all children have proper understanding, which contribute to preventing prejudice, discrimination, or stigmatizing about tungiasis and persons affected by it. Through it, we tried to minimize the mental burden. Also, confidentiality was maintained throughout the study. The treatment was applied to the lesions to be studied and other identified lesions. Also, for the excluded participants, we treated them with the treatment that works best when the intervention is finished.

In addition to monitoring the data and safety, an independent monitor oversaw this study. The monitor confirmed the appropriate implementation of the study and data reliability. Informed consent was obtained from all participants. Children and caregivers provided written assent/fingerprint and consent to participate in this study.

### Trial design

This study was a randomized, observer-blinded, parallel-treatment cohort trial with the intent of investigating the cure rates of the two treatments within 7 days. All participants received both treatments, 5% sodium carbonate, a local treatment method, and dimeticone (NYDA), the WHO standard treatment, one on each foot. Therefore, assignment to treatment groups, with participant and intervener masking, was not possible.

### Study period

The data collection period was from January 27, 2023, to February 28, 2023, during the dry season, when the prevalence of tungiasis is known to be at its peak and at a time not affected by holidays.

### Study population

The study recruited children aged 3–13 years living in the Suba-South and Ndhiwa sub-counties in Homa Bay County, Kenya. Children were included if diagnosed as affected with *Tunga penetrans* and manifesting the symptoms and signs induced by tungiasis, identified as Fortaleza classification stage II or III. Stage II, the early lesion, showed a brownish/black dot with a diameter of 1–2 mm surrounded or not by erythema, with or without local pain and itching). Stage III, the mature egg producing flea, had a circular yellow-white watch glass-like patch with a diameter of 3–10 mm and a central black dot (viability signs continue to show clear, intermittent egg expulsion, and rim forms around abdominal cone and a concave surface) [3], using a digital handheld microscope.

### Eligibility criteria

1. Children aged 3–14 years infected with tungiasis in Homa Bay, Kenya.
2. Children and caregivers provided written assent/fingerprint and consent to participate in this study.
3. Children with more than one viable flea in both feet, and 1–5 embedded fleas from both feet were enrolled.

### Exclusion criteria

1. Children infected with >20 fleas.
2. Children/caregivers declined to participate.
3. Children with another pruritic skin condition on their feet.

### Study area

The study was conducted in five villages in the Suba-South and Ndhiwa sub-counties, Homa Bay County, in western Kenya: Kisaku in Suba-South and Ramoyo, Kombe, Osogo, and Kaumo in the Ndhiwa sub-county. The total population of these areas are 343,074, and the prevalence of tungiasis in these two sub-counties was 1.1% (JICA grassroots project survey, 2022).

### Outcome measures

**Fig 1:**
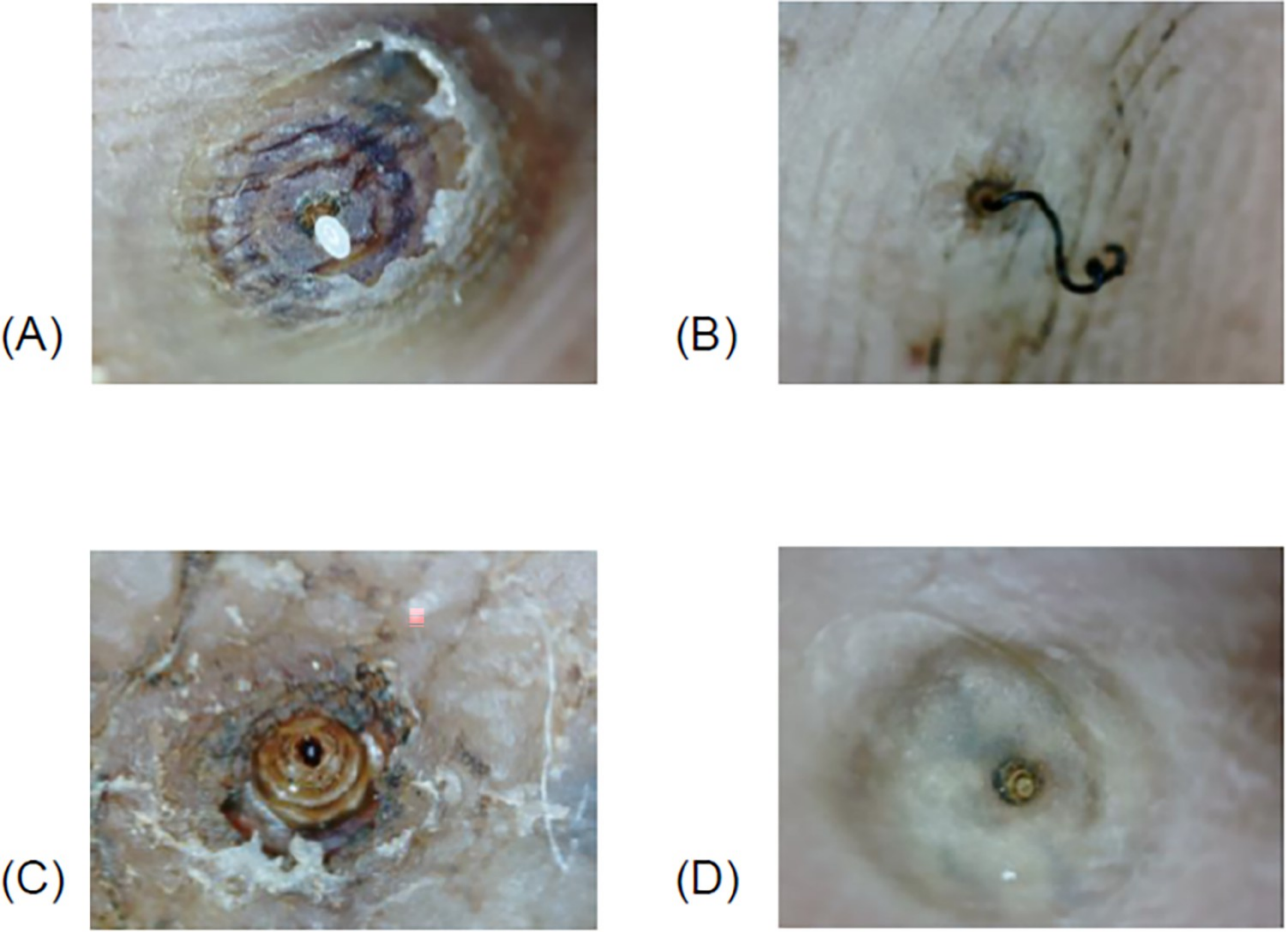
The four viability signs. Videos were taken with a handheld digital video microscope with a 5 mega-pixel optical resolution. (A) Expulsion of eggs, (B) excretion of fecal thread, (C) excretion of fecal liquid, and (D) pulsations/contractions. For (D), the color of the surrounding skin changed rhythmically with the beating of the pulse. Also, it is possible to see the external, moving abdominal cone.

The primary endpoint was the cure rate (proportion of dead fleas) on day 7 after treatment with either 5% sodium carbonate solution or NYDA. Embedded fleas were assessed using a digital handheld microscope at baseline and on days 3, 5, and 7 for the following four signs of viability: expulsion of eggs, excretion of fecal threads, excretion of fecal liquid, and pulsations/contractions (Fig 1).

An embedded flea was considered nonviable when none of the four viability signs were detected during the 15 min of observation.

The secondary endpoints were adverse effects (nausea/vomiting, arthralgia, fever, headache, fatigue/malaise, myalgia, and chills), acute pathology scores (pain, itching, erythema, warmness, edema, desquamation, fissure, suppuration, ulcer, abscess, and lesions in clusters), and differences in feelings of children during the 7 days after treatment as well as easiness of treatment according to parents. Each participant was asked to rate their experience of pain and itching using a visual analogue scale with 4 4-stage assessment [16], and an inflammation score was used for this assessment by trained nurses/ clinical officers (COs) [14]. This inflammation score [14] was based on one foot, one foot separated into nine areas, and each inflammation presence scores in a range of 0.5–3.0 points.

### Sample size

We randomized patients into different arms to limit bias and estimated the required sample size based on the estimation of treatment efficacy with precision. The sample size was estimated to have an 80% efficacy rate, with an absolute precision of 10%; therefore, 62 lesions were enrolled in each concurrent parallel treatment cohort.

### Sampling and recruitment

Sampling and recruitment were conducted in early childhood development and public primary schools that reported cases from community health promoters (CHPs). In the study area, there were 275 public schools. Children whose skin conditions were suspected by the CHPs to represent tungiasis were screened for eligibility to participate in this study. The children and their caregivers, someone who takes care of a child (parent/guardian/head of household), were then asked for their formal written (including thumb stamp) assent and consent after the study objectives and procedures were explained to them. Verbal consent was not allowed. If one refused to assent/consent, it was planned that the child would not be included in the study, but there were no such cases. As there were few cases from each school, we recruited all candidates reported by the CHPs. We sampled 1–5 embedded lesions per foot that were not clustered, in a right-to-left, 1:1 ratio, using the leg with fewer lesions. According to the Fortaleza classification [3], the growth level of the target lesions was stage II or III. Only lesions that could be clearly distinguished from one another were included in this study. The children were instructed not to manipulate the lesions for the next 7 days.

### Randomization, allocation, and blinding

All study participants received 5% sodium carbonate and NYDA, with one treatment per foot. At the beginning of the study, a sealed envelope was used to randomize the two treatments. The sealed envelopes contained either of the following written notes with a participant number: “Right foot: dimeticone, Left foot: 5% sodium carbonate” or “Right foot: 5% sodium carbonate, Left foot: dimeticone”. Blinding of the research assistant administering the treatment was not possible because 5% sodium carbonate required soaking of the foot, whereas NYDA was directly applied to each lesion. All observers were blinded to the study protocol. To reduce the risk of detection bias, the observer was the sole investigator.

### Intervention

The intervention was performed by trained nurses or COs on day 1 only. After washing the affected area with soap and water, both treatments were performed, with one treatment on each foot. One foot was soaked in 5% sodium carbonate solution. The solution was prepared by dissolving 250 g of sodium carbonate in 5 L of warm potable water in a deep basin. The temperature of water was set at 35–40°C. The affected parts were soaked in the solution for 15 min. Although sodium carbonate is water soluble, it dissolves quickly and is set at a temperature that is comfortable enough for the individual to insert their feet for 15 minutes. The temperature was set such that it would not change the effect, as described in a previous study [13]. The other foot was treated with NYDA for each lesion. In a study that examined the application method using NYDA, the targeted application killed embedded fleas more rapidly than when the entire foot was covered [10]. Thus, this method of application to the target area was used in the present study. Three drops were directly applied to each target lesion. One drop corresponded to 0.05 mL of NYDA, and the procedure was repeated thrice within 10 min to ensure that the maximum amount of NYDA entered the abdominal cone of the parasite within a short period. Thus, approximately 0.15 mL per lesion was required. After the treatment and drying, clean socks and new sandals were provided to cover the feet.

### Monitoring

Monitoring was simultaneously performed on days 1, 3, 5, and 7. Trained nurses or COs monitored the participants, and interviews were conducted under the guidance of a doctor at treatment initiation and final decision. They started by examining their vital signs and moved to check the adverse effects, acute pathology, and differences in feelings of children during the 7 days after treatment as well as the easiness of treatment according to parents. Patients’ experiences of itching and pain in each foot were assessed according to the following scores of itching or pain: 0, none; 1, a little; 2, quite a lot; and 3, very much. The inflammation Score was used according to Thielecke’s development [14]. The highest Total Inflammation Score was 27.

### Adverse reactions and events

The study participants were carefully examined on days 1, 3, 5, and 7 and were asked by the nurses or COs about adverse reactions such as blister, soreness, fever, and headache. During each examination, the participants’ temperature, blood pressure, and pulse rate were recorded. Adverse events were recorded in the adverse events log.

### Statistical analyses

As the primary outcome of this study was binary categorical data that recruited multiple lesions per person, a generalized linear mixed-effects model was used to analyze the paired data, with the population of individuals included as random factors. The per-protocol set was adopted in this study to compare local practices with the standard WHO-recommended treatment. Only treatment-adhered study participants were analyzed, and instances of flea removal were excluded such that each participant had no missing values for flea infections. The log-rank test was performed to compare the trend of non-viability rate of the two treatments. The Wilcoxon signed-rank test was performed to compare the differences in terms of safety of both treatments. All data were analyzed using EZR version 1.54, which is a graphical user interface for R version 4.0.3. More precisely, it is a modified version of R commander designed to add statistical functions frequently used in biostatistics [17].

## Results

### Pre-study

The preliminary study assessed the safety of >95% pure sodium carbonate (industrial grade; bought from Tata Chemicals Magadi Limited), including inflammation, after its application to the healthy skin of the feet of 10 adults. Informed consent was obtained from all participants. Because the previous study was also performed with the same product, as anticipated, the present study with 5% sodium carbonate did not show changes in vital signs during or after treatment. Two participants mentioned slight itchiness while soaking their feet in the 5% sodium carbonate solution, but no pain was reported. No acute pathologies were observed.

### Environment

The target schools were at altitudes of 1274 to 1679 m (median, 1415 m), the temperatures of the study sites ranged from 22.7°C to 35.6°C (median, 30.5°C), and the humidity varied from 29 to 43% (median, 37%). There were no rainy days during the data-collection period.

### Participants

**Fig 2:**
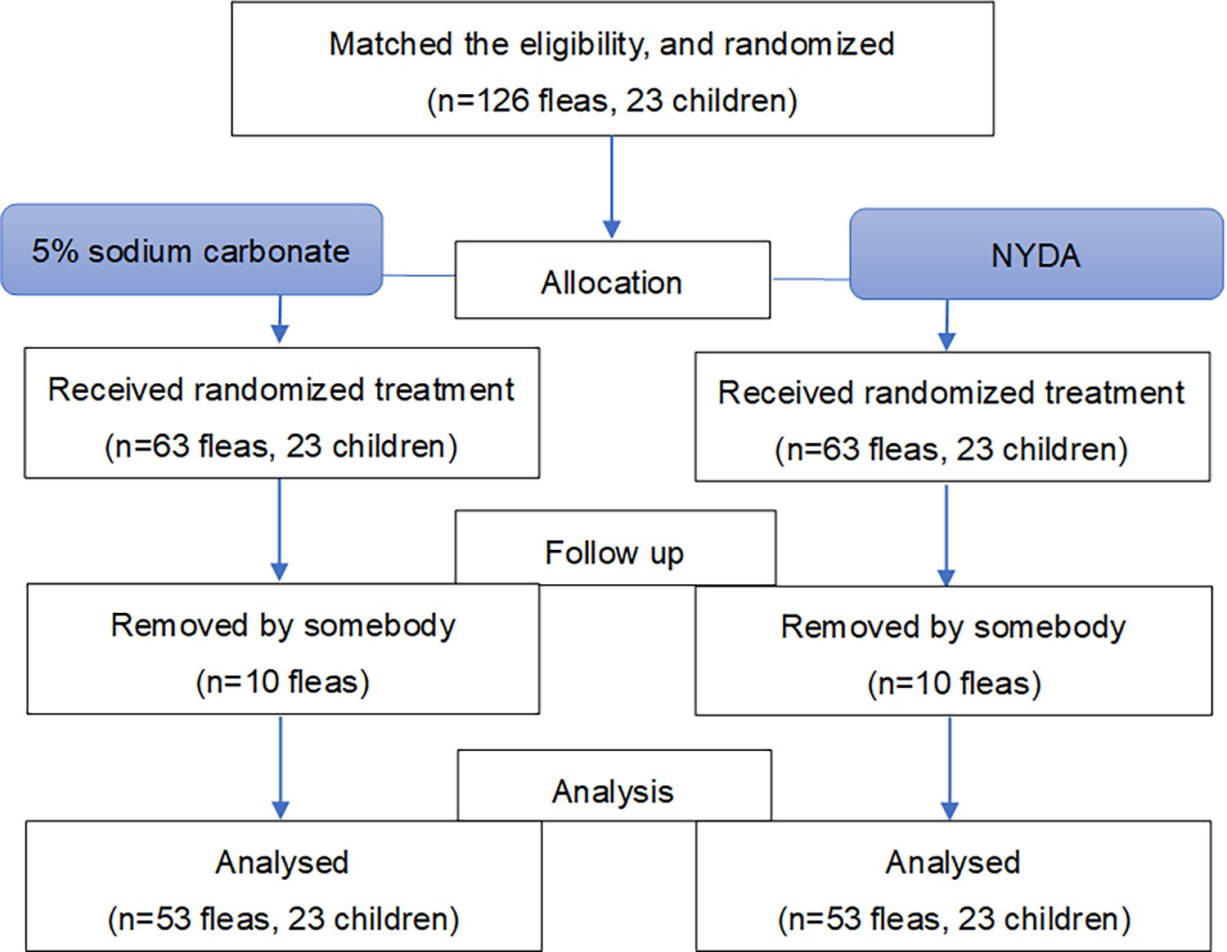
Flow diagram of assessed/intervened/analyzed fleas through the trial.

In total, 126 flea infections in 23 participants were described during the initial screening, but only 106 were included in the final analysis after excluding 20 cases of flea removal (Fig 2). All 23 children who underwent randomization completed the study. The male-to-female ratio of the participants was 57–43%, and children aged 3–13 years were recruited. The most frequent type of footwear was open shoes, and the most frequent type of school uniform was partially owned; more than half of the participants (61%) accounted for each of these results. All participants answered that they slept on the floor at home. When asked about their knowledge of tungiasis, 83% answered correctly about the causes, and 100% answered correctly about the symptoms (Table 1).

**Table 1:** Characteristics of the participants.

		Total (n = 23)	
Children		n	%
Sex	Male	13	57%
	Female	10	43%
Age	3-5yrs	6	26%
	6-8yrs	7	30%
	9-13yrs	10	43%
Foot wear (at the baseline)	None	3	13%
	Open	14	61%
	Closed	6	26%
Presence of school uniform	None	7	30%
	Partially	14	61%
	Complete	2	9%
Sleeping place	Bed	0	0%
	Floor	23	100%
Knowledge on the cause of tungiasis	Correct (fleas)	19	83%
	Incorrect (soil)	4	17%
Knowledge on the symptoms of tungiasis	Correct	23	100%
	Incorrect	0	0%
		**Total (n = 11)**	
**Head of the Household**		n	%
Age	20’s	3	27%
	30’s	6	55%
	40’s	2	18%
Occupation	Farmer	11	100%
Knowledge on the symptoms of tungiasis	Correct	9	82%
	Incorrect	2	18%
Thought or idea of tungiasis	Same as scratches	2	18%
	Slight injury	1	9%
	Rare disease	0	0%
	Serious disease	8	73%

We involved 11 heads of households since Some children were siblings. All heads of the households in the study held farming occupations, and 82% were in their 20s or 30s. We found that 82% of the patients understood the symptoms of tungiasis, and 73% considered it a serious disease (Table 1).

### Cure rates (percentage of dead fleas)

On the day 7, the proportion of dead fleas treated with 5% sodium carbonate was 64%, and the proportion of dead fleas treated with NYDA was 87% (Table 2). The generalized linear mixed-effects model results showed a significant difference between the 5% sodium carbonate and NYDA treatment groups (*P*<0.01). Subgroup analysis was performed as per age; specifically, children aged 8 to 13 years showed a significant difference between the 5% sodium carbonate and NYDA (*P* = 0.02) treatments, whereas children aged 3 to 7 years showed no significant difference (*P* = 0.14). The 3–7 years age group showed a higher percentage of responses to flea death than the 8–13 years age group, especially those treated with 5% sodium carbonate.

**Table 2:** Primary outcomes.

	5% sodium carbonate	NYDA	Estimate	SE	*P*	OR
	Total no. of respondents (%)	Total no. of respondents (%)				
**Primary outcome**						
Total number of fleas alive	19 (35.8)	7 (13.2)	1.67	0.59	<0.01	5.32
Total number of fleas dead	34 (64.2: 95%CI 49.8–76.9)	46 (86.8: 95%CI 74.7–94.5)				
Total number of fleas removed	10	10				
**Sub-group**						
3-7yrs number of fleas alive	7 (24.1)	3 (10.7)	1.30	0.89	0.14	3.67
3-7yrs number of fleas dead	22 (75.9)	25 (89.3)				
8-13yrs number of fleas alive	12 (50)	4 (16)	1.88	0.78	0.02	6.58
8-13yrs number of fleas dead	12 (50)	21(84)				

**Fig 3:**
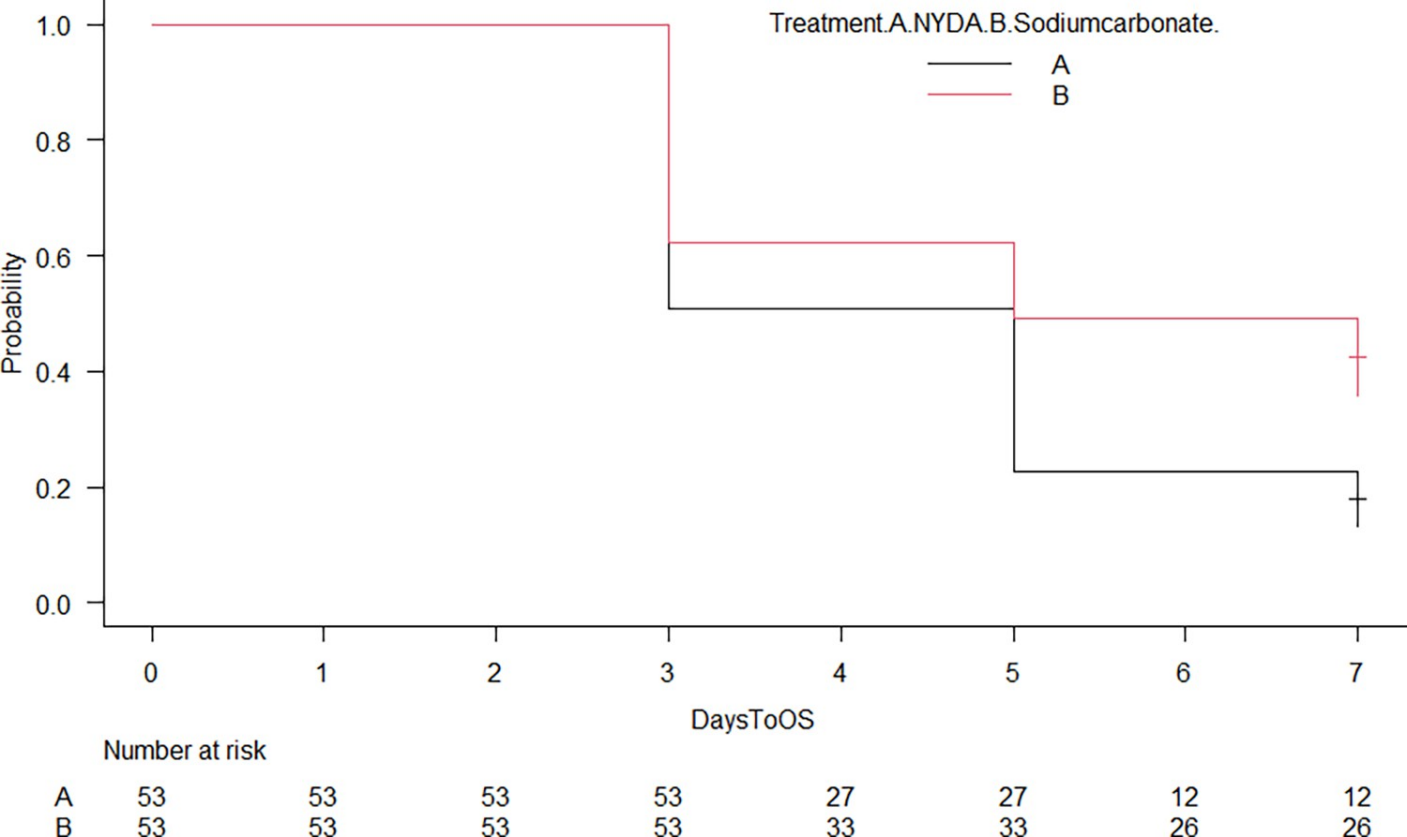
Kaplan-Meier plot of fleas remaining alive by time point.

Fig 3 shows the Kaplan-Meier plot of fleas remaining alive by time point. Median survival was 5 days for both treatments; NYDA had a significantly higher trend of flea non-viability rate than 5% sodium carbonate (*P*<0.01).

Moreover, 20 fleas (NYDA: 10 fleas; 5% sodium carbonate: 10 fleas) were excluded from the analysis (Fig 1), because we did not know whether they died or were removed by someone else.

### Safety

**Fig 4:**
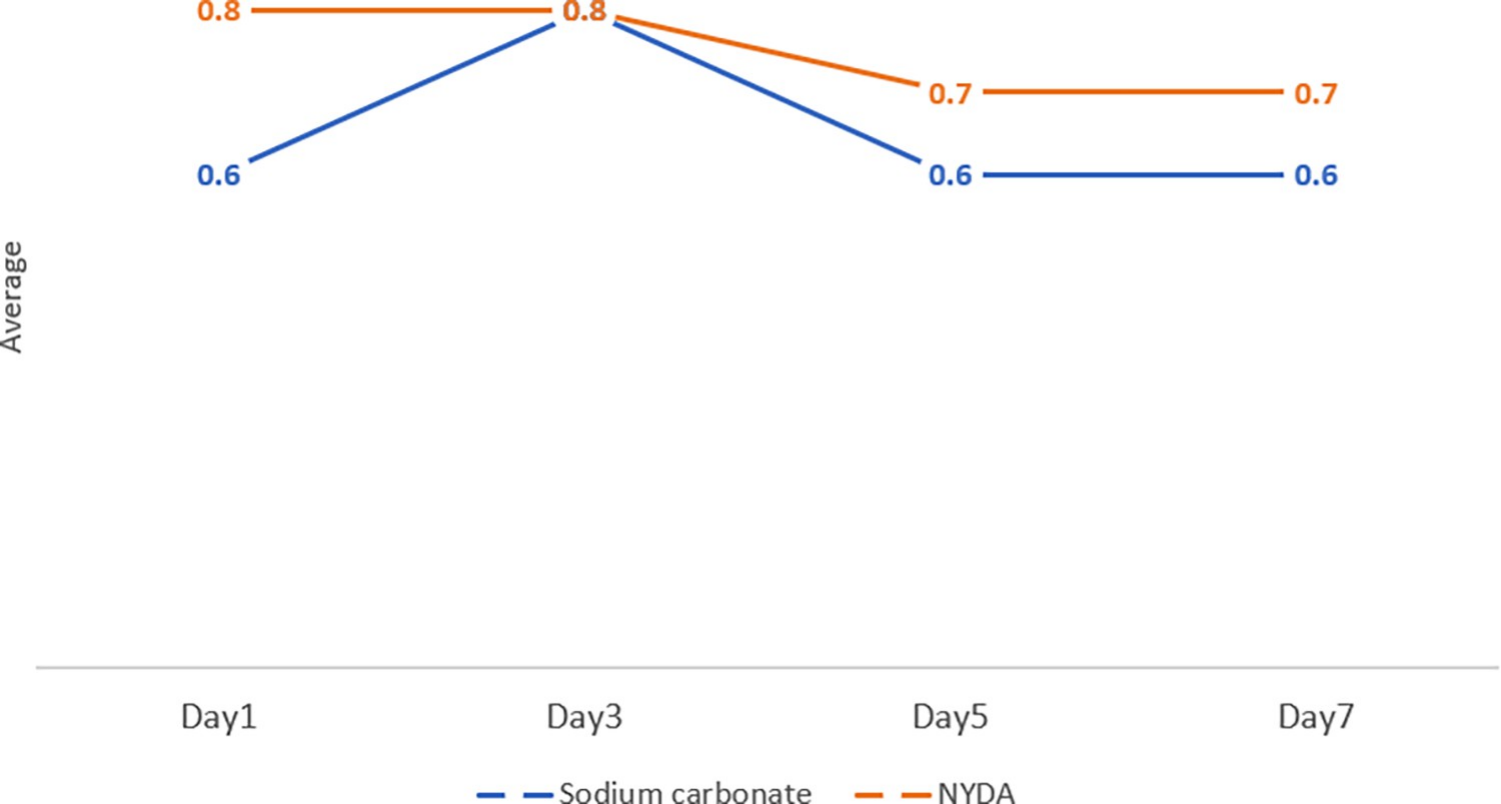
Inflammation score.

Changes in the average inflammation scores are shown in Fig 4. NYDA treatment resulted in a slow decline in inflammation scores, whereas the inflammation scores peaked on day 3 after sodium carbonate treatment. There were no significant differences in either score. Table 3 shows the assessment of pain and itchiness during the 7-day period. Most of the participants answered that they experienced no differences during this term. However, only one case of each treatment for pain and two cases of sodium carbonate treatment for itchiness showed an increase on the scale. Five participants had temperatures over 38°C at some point during the monitoring period, and one participant had a cough. All participants immediately consulted a medical doctor, and in all instances, the doctor confirmed that the condition of the participants was not related to the study. All were administered the medication, and the fever disappeared the following day, and other events did not occur.

**Table 3:** Cases of pain and itchiness.

	NYDA	Sodium carbonate
**Pain**		
No. of increased cases	1	1
No. of decreased cases	3	4
No difference	19	18
**Itchiness**		
No. of increased cases	0	2
No. of decreased cases	1	8
No difference	22	13

There was no decision to terminate the study because serious adverse effects/events did not occur.

### Feelings, ease, and participant choice of favorite treatment

**Fig 5:**
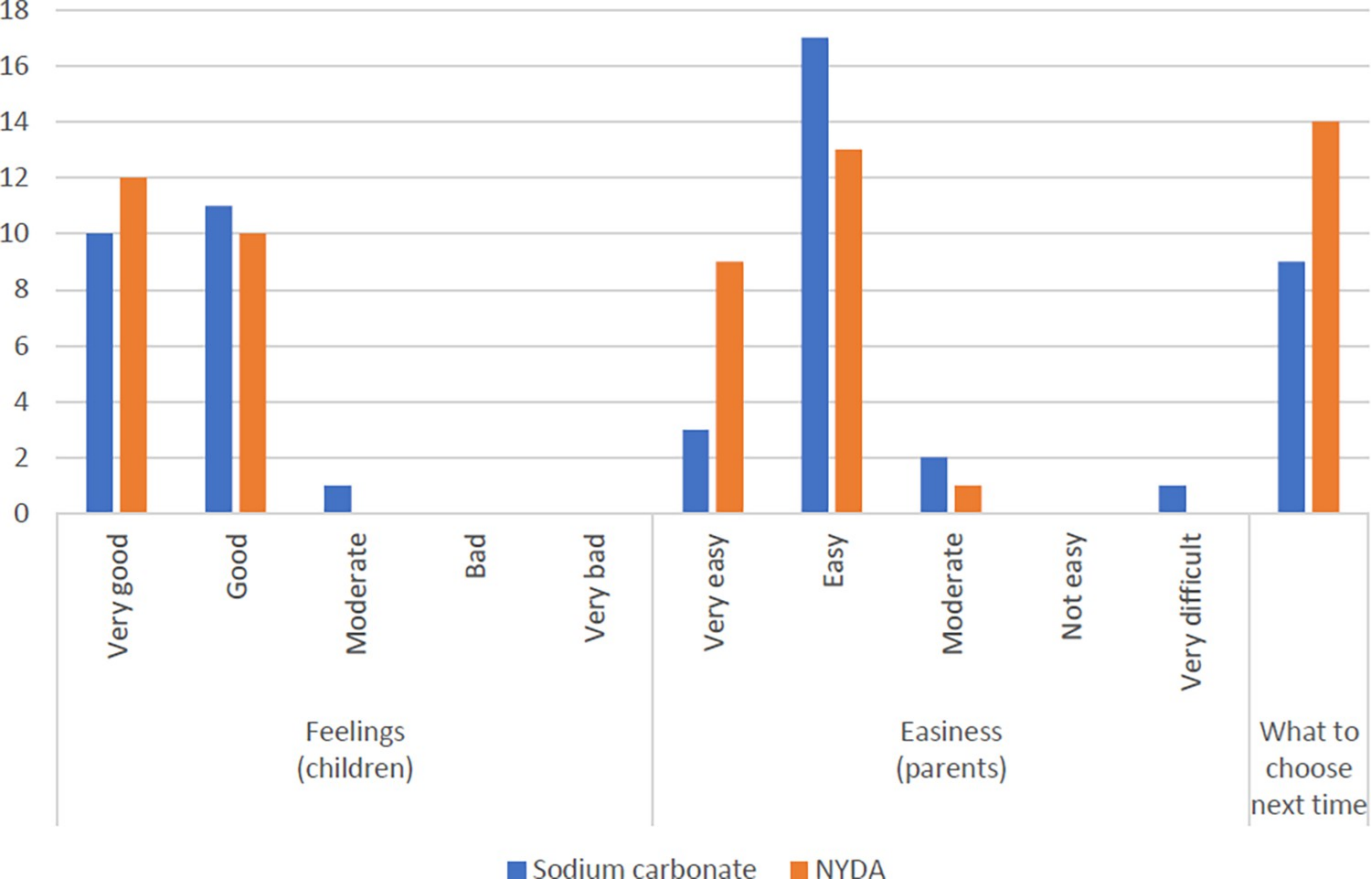
Feelings, easiness, and favorite during tungiasis treatment.

The children were asked to describe their feelings during treatments, and the parents were asked about their possible ease of in using these treatments (Fig 5). Twenty to twenty-two children and their parents answered that their feelings were very good/good and that both treatments were very easy/easy to use. Only one parent stated that sodium carbonate was very difficult to use because its costs were unaffordable. On the last day, 14 children answered that they wanted to select NYDA for possible future treatments, whereas nine children chose sodium carbonate.

## Discussion

Because of the expense and unavailability of the WHO-recommended treatment for tungiasis, NYDA, in Western Kenya, we investigated the efficacy of a local treatment of soaking infected feet in a 5% sodium carbonate solution. We found that this treatment was 64% effective within 7 days and approximately 23% less effective than NYDA (87%).

### Future research should compare cure rate and safety

The efficacy obtained in this study matched that of previous clinical trials [10,13,14]. However, it has a variation with Nordin’s study with an 97% cure rate of NYDA [10]. It may be because of the observer’s blinding since the study by Nordin was not blinded (no indication). The cure rate of 5% sodium carbonate on day 7 was inferior to that of NYDA; it was significantly lower than that of NYDA. However, 5% sodium carbonate showed a greater effect with respect to the cure rate on day 7 than potassium permanganate (39–40% [9,14]), which is the most frequently used comparison treatment in previous studies on tungiasis. When we compared the trend of non-viability rate during the 7-day observation, median survival was 5 days for both treatments (Fig 3); future studies should consider expanding the observation period to see their dead flea percentage movement longer. Neem oil has been shown in another study [9] to have a delayed impact on viability signs of fleas, interfering with the normal development of insects [18]. In addition, the results showed that the mortality rate of the two treatment methods was reversed depending on the location (S4 Data). Since it is possible that the character may differ depending on the type of gene in the *tunga penetrans*, better results would be obtained if gene mapping could be conducted at the same time in the future.

**Fig 6:**
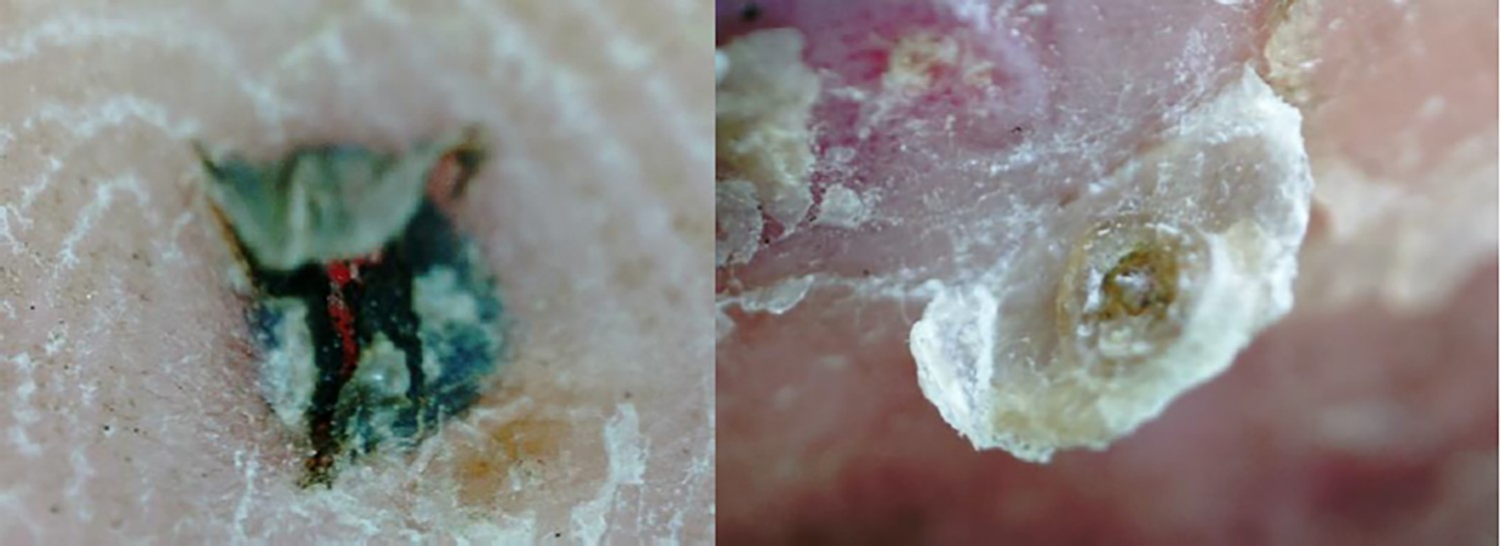
Picture of the skin at the location of a removed flea. Left, self-removed; Right, skin dropped with flea.

Although the participants were requested not to remove the fleas, there were 20 such instances (Fig 6), likely in an attempt to alleviate pain and itching. There was no difference with respect to the foot treatment and flea removal—specifically, 10 fleas were removed during NYDA treatment and 10 fleas were removed during 5% sodium carbonate treatment—the different treatments did not influence the urge to self-remove fleas.

### The possibility of new observation and evaluation methods to assay the absence of fleas

We found a possibility of shortening the observation time for viability signs. Although the observation time was set to 15 min, viability was confirmed within 8 min in all cases. A 10-min observation time was cited in a previous study [9]; however, the assay length was unknown for other clinical studies. In future studies, it would be prudent to consider shortening the observation time for viability signs to reduce the patient burden.

It was difficult to differentiate the reason of the pain and itchiness between because of flea infections, owing to the side effects of the treatment products. However, some methods can be used; for example, we can ask about stinging pain immediately upon the application of the product. Infrared thermography is another effective method for measuring inflammation [19]. Inflammation may initially increase in the week after treatment as the fleas die but should then decrease.

During this 7-day observation period, many children experienced new effects. Therefore, because of the possibility of pain and itching due to new lesions, we asked the patient to specifically point to the part with a finger to know that recruited lesions’ pain and itching.

Younger participants aged 3–4 years, did not uniformly give informative replies to questions; therefore, future research should restrict the participant pool to children aged ≥5 years.

### Mechanism of action and safety of sodium carbonate

The mechanism of action of sodium carbonate solution on tungiasis could not be investigated in this study. No study tried soaking in plain water; however, if we compare it with the study that used KMnO4 solution [9,14], the cure rate is around 30% different with this sodium carbonate solution. Therefore. We can say it is not only because of the water suffocation. Ambenje and Otieno [13] suggested possible osmotic effects or simple suffocation. However, it is difficult to imagine that fleas themselves are pushed out by osmotic pressure; therefore, it can be thought that aging hastens the effect. Sodium carbonate is strongly alkaline, softens protein on the skin surface, and emulsifies it with fat and waste products, making it easier to remove. It is also considered suitable for injuries because it increases skin metabolism. It can be assumed that this type of action enters the qigong part of the flea, which is suffocated.

Redness is the only reported side effect of sodium carbonate on the skin [20]; however, redness was reported in seven participants in this study: both feet in one participant and one foot in six participants. Of the six participants who reported redness in only one foot, three were treated with NYDA, and the remaining three were treated with sodium carbonate. One of the study limitations is that we didn’t have the differentiation of the inflammation score used as a measure of safety or an indicator of the regression of inflammation following the death of the fleas. Another limitation is that some of the participants are siblings (five siblings from a family at most). Thus, the potential for data bias might be there since the total number of children is also a few. For future studies, we need to add measures to differentiate them and consider to limit the number of siblings recruited from a family.

The sodium carbonate used in this trial had higher purity than that available in a local market in Kenya. Therefore, we cannot recommend the community to use sodium carbonate from the market, mainly because of its safety. However, findings from this study suggest the further study to confirm sodium carbonate’s effectiveness and safety for the treatment of tungiasis.

### Conclusion

From the Pilot study, we conclude that NYDA was significantly more effective than 5% sodium carbonate for tungiasis treatment. Both treatments were safe, but the participants had more preference for NYDA. Future studies should be conducted and consider having a shorter observation time of viability of *tunga penetrans* and more days of observation to confirm the efficacy and safety of local practices in comparison with other treatments.

## Supporting information

S1 CONSORT Checklist CONSORT 2010 checklist of information to include when reporting a randomized trial. (PDF)

S1 Data Primary outcomes. (XLSX)

S2 Data Pain and itching. (XLSX)

S3 Data Acute pathology. (XLSX)

S4 Data Cure rates differences in different areas of the study region. (DOCX)
