# Supplementary material for: A pilot study of dimeticone oils versus sodium carbonate treatment for tungiasis: A randomized cohort trial in Homa Bay County, Kenya
Source: PLoS Negl Trop Dis. 2024 Jul 23;18(7):e0012341. doi: 10.1371/journal.pntd.0012341 (PMC11296639; doi:10.1371/journal.pntd.0012341)
Supplement: S4 Data — (DOCX) [file pntd.0012341.s005.docx]

S4. Cure rates differences in different areas of the study region.
